# Letter to the Editor: “Parietal Cell Antibody Levels Among Chronic Gastritis Patients in a Country With Low *Helicobacter pylori* Infection: Epidemiology, Histopathological Features, and *H. pylori* Infection”

**DOI:** 10.1111/hel.70053

**Published:** 2025-06-03

**Authors:** Xiaopei Guo, Manon C W Spaander, Gwenny M Fuhler

**Affiliations:** ^1^ Department of Gastroenterology and Hepatology Erasmus MC Rotterdam the Netherlands

To the Editor,

We read with great interest the article titled “Parietal Cell Antibody Levels Among Chronic Gastritis Patients in a Country with Low *Helicobacter pylori* Infection: Epidemiology, Histopathological Features, and *H. pylori* infection” by Amalia et al. [1]. In this study, the authors investigated the prevalence of autoimmune gastritis (AIG), and its association with *Helicobacter pylori* ( *H. pylori* ) infection status and gastric histopathological features in the Indonesian population. Given the previously reported high incidence of gastritis and relatively low *H. pylori* infection rates in this region, exploring other causes of gastritis, such as AIG, is essential for improving diagnostic accuracy and guiding clinical risk management [2].

While the authors conducted a comprehensive analysis of parietal cell antibody (PCA) levels and its association with clinicopathological features, several aspects of the study's methodology and interpretation raise concerns and warrant further clarification. A key observation of this study is the reported 78.99% prevalence of PCA positivity among 113 Indonesian patients with chronic gastritis and 25 healthy controls, while only one patient was diagnosed with AIG. The authors suggest that this discrepancy might be due to *H. pylori* ‐associated immune activation. However, we think it may also reflect the combined impact of overly strict diagnostic criteria for AIG and the inappropriate interpretation of PCA results.

In the diagnostic methods, AIG was defined based on the PCA positivity, absence of *H. pylori* infection, histopathological features associated with AIG, and sparing of the antrum. We understand the concern that coexisting *H. pylori* infection may complicate the diagnosis of AIG, as it can cause histopathological changes that resemble AIG. But we note that neither antrum sparing nor negative *H. pylori* ‐negative status should be a required criterion for AIG diagnosis. Our previous findings have shown that AIG can coexist with *H. pylori* infection, and hence patients may present with antral inflammation or atrophy. Such patients still show characteristic serological features similar to AIG patients without *H. pylori* infection, including decreased pepsinogen (PG) I levels and elevated gastrin 17 [3]. Additionally, they may exhibit pathological findings such as enterochromaffin‐like (ECL) cell hyperplasia [4], which was not investigated in the study by Amalia et al. Therefore, we think the criteria used may have led to underdiagnosis of AIG in the Indonesia cohort. Rather than excluding patients with *H. pylori* infection or antrum inflammation/atrophy, more specific diagnostic tests should be considered to improve the diagnosis of AIG, especially given this is the first study reporting AIG prevalence in this region.

Adding to these diagnostic challenges Beyond these diagnostic criteria, the method used for detecting PCA in the study also needs careful consideration. The presence of PCA was detected by Amalia et al. using an indirect immunofluorescence antibody test (IFT), a semi‐quantitative method which has high sensitivity but limited specificity. Notably, nearly half of the patients had borderline PCA levels (10 units/mL, in an assay ranging from 0 to > 300 units/mL, with 10 units/mL being the first discrete value), which warrants careful interpretation. Our own studies showed that 81 of 256 (31.6%) patients tested positive for PCA using IFT, but only 18% were confirmed positive by the H^+^/K^+^‐ATPase‐specific EliA, an automated quantitative enzyme fluoroimmunoassay, and pathology [3]. Moreover, studies showed that PCA is present at lower rates in the general healthy adult population, who may never develop AIG or pernicious anemia [3, 5]. While Amalia et al. showed a correlation between gastric body inflammation score and PCA levels, it is unclear from their report whether controls exhibited a lower prevalence of PCA compared to gastritis patients. Thus, additional tests may be required to prove the presence of PCA.

The authors suggest a potential link between the high PCA prevalence observed in this cohort and *H. pylori* infection. While the hypothesis that *H. pylori* could trigger autoimmune responses via molecular mimicry is biologically plausible, the current PCA data presented in this study may be somewhat limited in supporting this interpretation with confidence. To explore this further, we examined data from 81 patients with gastric atrophy and found no significant differences in PCA levels (determined by EliA) between patients with and without *H. pylori* infection (36.1 vs. 39.4, *p* = 0.24, Figure 1A). Further correlation analysis showed that the height of the serum level of PCA is not associated with the *H. pylori* antibody titer in patients with *H. pylori* infection (*r* = 0.08, *p* = 0.66, Figure 1B).

**FIGURE 1:**
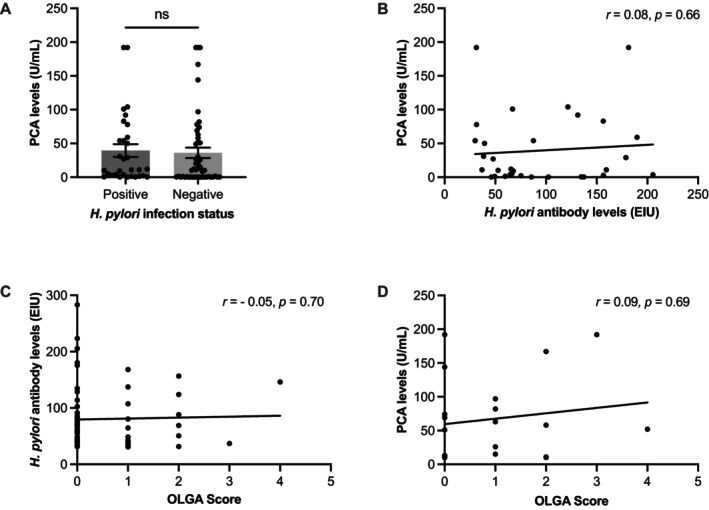
(A) Serum PCA levels (U/mL; cut‐off: 10 U/mL) in individuals with positive and negative *H. pylori* infection status. (B) Pearson correlation analysis indicates no significant association between PCA levels and *H. pylori* antibody titers (*r* = 0.08, *p* = 0.66). (C) Spearman rank correlation shows no significant relationship between *H. pylori* antibody levels and OLGA stage, representing overall atrophy severity (*r* = −0.05, *p* = 0.70). (D) In *H. pylori* ‐negative patients, Spearman rank correlation analysis reveals no association between PCA levels and OLGA stage (*r* = 0.09, *p* = 0.69). OLGA score, operative link on gastritis assessment staging system; PCA, parietal cell antibody. The assay methods and cut‐off values for PCA and *H. pylori* antibody levels are described in a previous study [3].

Finally, the authors examined *H. pylori* antibody levels and PCA levels in relation to clinicopathological features, such as inflammation, atrophy, and PG I/II ratio. Results of their study indicate that levels of *H. pylori* antibody and PCA were higher in patients with gastric antrum or body inflammation, as well as in those with atrophy. This seems expected given that *H. pylori* infection and AIG are known to cause inflammatory and atrophic mucosal changes. However, the height of antibody titers in *H. pylori* ‐infected patients and those with AIG may be affected by patient genetics, BMI, bacterial load, mounted immune response, antibody decay, and many other factors [6]. Thus, it remains unclear what a correlation between IgG levels and atrophy stage would biologically mean, and it would be of interest to see whether the correlation remains significant after the exclusion of *H. pylori* seronegative and PCA negative patients from their linear regression analyses. Our own data show that, at least in our cohort of patients with premalignant lesions of the stomach, the level of atrophy as determined by OLGA score is not correlated with IgG levels of anti‐ *H. pylori* antibodies (*r* = −0.05, *p* = 0.70, Figure 1C) or PCA (*r* = 0.09, *p* = 0.69, Figure 1D).

In summary, we think the diagnostic criteria used by Amalia et al. may have resulted in underestimation of AIG prevalence in the Indonesian cohort, while the prevalence of PCA may have been overestimated. More specific serological assays (e.g., EliA, anti‐intrinsic factor antibody, pepsinogen I, and gastrin‐17) and additional histological features like enterochromaffin‐like (ECL) cell hyperplasia should be incorporated to improve the detection of PCA and diagnosis of AIG.

## Author Contributions

**Xiaopei Guo:** conceptualization, writing original draft. **Manon C W Spaander:** conceptualization, review and editing. **Gwenny M Fuhler:** conceptualization, writing and editing.

## Disclosure

The authors have nothing to report.

## Ethics Statement

The study was approved by the Medical Ethical Review Committee of Erasmus MC (MEC‐2009‐090), and informed consent was obtained from all included participants.

## Data Availability

The data that support the findings of this study are available on request from the corresponding author. The data are not publicly available due to privacy or ethical restrictions.

## References

[1] R. Amalia , M. Miftahussurur , A. F. Syam , et al., “Parietal Cell Antibody Levels Among Chronic Gastritis Patients in a Country With Low *Helicobacter pylori* Infection: Epidemiology, Histopathological Features, and *H. pylori* Infection,” Helicobacter 30 (2025): e70035. 40249164 10.1111/hel.70035

[2] M. Miftahussurur , S. Shiota , R. Suzuki , et al., “Identification of *Helicobacter pylori* Infection in Symptomatic Patients in Surabaya, Indonesia, Using Five Diagnostic Tests,” Epidemiology and Infection 143 (2015): 986–996. 25034254 10.1017/S095026881400154X PMC9507110

[3] X. Guo , M. W. J. Schreurs , F. E. Marijnissen , et al., “Increased Prevalence of Autoimmune Gastritis in Patients With a Gastric Precancerous Lesion,” Journal of Clinical Medicine 12, no. 19 (2023): 6112. 37834796 10.3390/jcm12196152 PMC10573100

[4] X. Guo , M. C. W. Spaander , and G. M. Fuhler , “Enterochromaffin‐Like Cell Hyperplasia as Identification Marker of Autoimmune Gastritis in Patients With *Helicobacter pylori* Infection in the Context of Gastric Premalignant Lesions,” Archives of Pathology & Laboratory Medicine 146 (2022): 1181–1182. 36174199 10.5858/arpa.2022-0097-LE

[5] E. Rusak , A. Chobot , A. Krzywicka , and J. Wenzlau , “Anti‐parietal cell antibodies ‐ diagnostic significance,” Advances in Medical Sciences 61 (2016): 175–179. 26918709 10.1016/j.advms.2015.12.004

[6] S. Y. Lam , M. C. Mommersteeg , B. Yu , et al., “Toll‐Like Receptor 1 Locus Re‐Examined in a Genome‐Wide Association Study Update on Anti‐Helicobacter Pylori IgG Titers,” Gastroenterology 162, no. 6 (2022): 1705–1715. 35031300 10.1053/j.gastro.2022.01.011 PMC11734630

